# Detection and treatment of peritoneal metastases in colon cancer: COLOPEC trial compared to routine practice

**DOI:** 10.1111/codi.70614

**Published:** 2026-09-17

**Authors:** Iris Hochstenbach, Julie J. M. Hamm, Eva Rademaker, Arend G. J. Aalbers, Djamila Boerma, Wilhelmina M. U. van Grevenstein, Patrick H. J. Hemmer, Ignace H. J. T. De Hingh, Niels F. M. Kok, Jurriaan B. Tuynman, Eva V. E. Madsen, Johannes H. W. de Wilt, Esther C. J. Consten, Henderik L. van Westreenen, Pieter J. Tanis, the COLOPEC Collaborators Group, Dutch Snapshot Research Group and Dutch Complex Colon Cancer Initiative, Caroline S. Andeweg, Vivian P. Bastiaenen, Willem A. Bemelman, Jarmila D.W. van der Bilt, Johanne Bloemen, Frank C. den Boer, Daan ten Bokkel Huinink, Cancer Clinic, Alexandra R.M. Brandt‐Kerkhof, Andre J.A. Bremers, Walter J.A. Brokelman, Huib A. Cense, Geert‐Jan Creemers, Johannes Crezee, Rogier M.P.H. Crolla, Jan‐Willem T. Dekker, Jennifer Demelinne, Marc J. van Det, Ziekehuisgroep Twente, Karin K. van Diepen, Marjolein Diepeveen, Gastrointestinal Surgery, Marcel G.W. Dijkgraaf, Eino B. van Duyn, Medisch Spectrum Twente, Esther D. van den Ende, Ziekehuisgroep Twente, Pauline Evers, Hans F.J. Fabry, Floris T.J. Ferenschild, Maashospital Pantein, Sebastiaan Festen, Onze Lieve Vrouwen Gasthuis, Anna A.W. van Geloven, Erwin van der Harst, Jeroen Heemskerk, Joost T. Heikens, Daniel A. Hess, Bas Inberg, Streekziekenhuis Koningin Beatrix, Jan Jansen, Charlotte E.L. Klaver, Frank W.H. Kloppenberg, Treant Zorggroep, Thomas J.M. Kootstra, R. T. J. Kortekaas, Maartje Los, H. C. J. van der Mijle, Linda Mol, Gijsbert D. Musters, Peter A. Neijenhuis, Simon W. Nienhuijs, Loes van den Nieuwenhof, Koen C.M.J. Peeters, Sebastiaan W. Polle, Jolien Pon, Pieter Poortman, Cornelis J.A. Punt, Sandra A. Radema, Bert van Ramshorst, Philip R. de Reuver, Koen P. Rovers, Roderick F. Schmitz, Lotte Schoonderwoerd, Nina Sluiter, Dirkje W. Sommeijer, Eric Sonneveld, T. C. van Sprundel, Sanne C. Veltkamp, Maarten Vermaas, Victor J. Verwaal, Emma Wassenaar, Johannes A. Wegdam, Marinke Westerterp, Marinus J. Wiezer, Daniel D. Wisselink, Fennie Wit, Arjen J. Witkamp, Karlijn van Woensdregt, Edwin S. van der Zaag, David D.E. Zimmerman, Mandy Zournas, Annette van Zweeden, D. C. Van der Aa, S. M. Van Aalten, I. Aanen, F. J. Amelung, Den Bosch, P. Van Amstel, E. Ancion, J. J. Atema, T. S. Aukema, V. Baart, E. Z. Barsom, V. P. Bastiaenen, T. Bazuin, M. A. J. Becker, V. M. Belvroy, R. H. A. Berndsen, B. Biersteker, J. D. W. Van der Bilt, J. G. Bloemen, S. Bluiminck, F. C. Den Boer, E. G. Boerma, M. C. Boonstra, W. A. A. Borstlap, L. A. Brugts, J. W. A. Burger, T. A. Burghgraef, S. M. M. De Castro, M. Cleveringa, R. J. S. Coelen, A. Demirkiran, E. Dijkema, S. Dijkink, S. A. Dingemans, L. Van Dullemen, E. B. Van Duyn, A. J. Eleveld, H. A. Galema, E. G. M. Van Geffen, R. T. J. Geitenbeek, A. A. W. Van Geloven, A. H. C. Gielen, C. A. Gispen, M. J. P. M. Govaert, V. P. Groot, B. A. Grotenhuis, A. A. J. Grüter, S. F. Hardon, A. G. Den Hartog, K. Havenga, J. A. G. Van der Heijden, F. Heilijgers, T. B. M. Van den Heuvel, R. S. Hijmans, J. Van Hilst, J. C. Hol, S. Janki, A. C. H. M. Jongen, I. Kappers, S. Karhof, B. A. J. Kertzman, M. M. Kieboom, N. Koemans, J. L. M. Konsten, W. H. Kopp, O. W. Kranenburg, P. Krielen, A. Kumas, M. Kusters, D. P. V. Lambrichts, B. Lamme, G. L. Van Leeuwen, E. W. Lockhorst, H. Lutfi, S. Malm, H. T. J. Mantel, G. A. Martini, P. Meijer, J. Melenhorst, D. M. Mens, MC Erasmus, M. J. H. Metman, L. C. F. De Nes, L. R. Moolenaar, J. A. Nieuwstraten, J. Nonner, S. E. Van Oostendorp, S. J. Oosterling, C. M. W. Pesch, C. M. L. Peters, M. W. H. Philipsen, W. Y. Van der Plas, M. M. Poelman, F. Polat, A. E. Posma‐Bouman, B. B. Pultrum, J. M. Van Rees, W. P. Reinders, P. R. De Reuver, M. C. Richir, T. W. H. Rijnhout, M. M. Romeijn, D. Roorda, C. C. Van Rossem, J. Rothbarth, M. Ruig, H. G. Sahin, I. Said, R. A. Schasfoort, J. Scholten, P. M. E. Schuivens, D. Schweitzer, C. Sietses, M. Sietzema, G. D. Slooter, N. Smakman, B. P. Smalbroek, E. J. Spillenaar Bilgen, E. J. A. Steller, T. F. Stoop, M. Straver, S. K. Stuart, A. Suntharan, A. K. Talsma, K. Trumpi, J. Veenhuizen, A. W. H. Van de Ven, E. G. G. Verdaasdonk, P. M. Verheijen, A. Villerius‐Meijer, M. Vissers, E. L. K. Voogt, R. P. H. De Vries, S. T. Van Vugt, D. D. De Waard, T. R. Wagner, A. K. Warps, R. H. A. Welling, M. Westerterp, J. K. Wiggers, A. R. Wijsmuller, C. D. M. Witjes, B. Zamaray, N. Van Zenden, S. J. M. Zomer, E. S. Zwanenburg, E. S. Zwart, S. L. M. Zwetsloot, M. J. Lahaye, P. D. Siersema, P. Snaebjornsson

**Affiliations:** ^1^ Department of Surgery Isala Zwolle the Netherlands; ^2^ Department of Surgery University Medical Centre Groningen Groningen the Netherlands; ^3^ Department of Surgical Oncology and Gastrointestinal Surgery Erasmus MC Cancer Institute Rotterdam the Netherlands; ^4^ Department of Surgery Antoni van Leeuwenhoek Hospital/the Netherlands Cancer Institute Amsterdam the Netherlands; ^5^ Department of Surgery St. Antonius Hospital Nieuwegein the Netherlands; ^6^ Department of Surgery University Medical Centre Utrecht Utrecht the Netherlands; ^7^ Department of Surgical Oncology Catharina Cancer Institute, Catharina Hospital Eindhoven Eindhoven the Netherlands; ^8^ GROW‐School for Oncology and Developmental Biology Maastricht University Maastricht the Netherlands; ^9^ Department of Surgery Amsterdam University Medical Centre Amsterdam the Netherlands; ^10^ Department of Surgery Radboud University Medical Centre Nijmegen the Netherlands; ^11^ Department of Surgery Meander Medical Centre Amersfoort the Netherlands

**Keywords:** adjuvant hyperthermic intraperitoneal chemotherapy, colon cancer, locally advanced colon cancer, peritoneal metastases, propensity score matching, routine practice, second‐look surgery

## Abstract

**Aim:**

The randomized COLOPEC trial demonstrated no benefit of adjuvant hyperthermic intraperitoneal chemotherapy (HIPEC) in preventing peritoneal metastases in locally advanced colon cancer. Because all patients underwent a diagnostic laparoscopy at 18 months, the study lacked a true control arm, leaving the effect of early detection and treatment compared with standard care uncertain. This study aimed to compare long‐term oncological outcomes between patients treated in the COLOPEC trial and patients treated in routine care.

**Method:**

Patients who underwent curative resection for c/pT4 or perforated colon cancer in the COLOPEC trial were compared with a retrospective control cohort that included all colon cancer resections performed in 2014–2015, in 50 Dutch hospitals. All 202 trial patients were matched 1:1 to 202 control patients using propensity score matching.

**Results:**

Following treatment in both arms of the COLOPEC trial, compared to the matched control cohort, 5‐year overall survival was 70.2% versus 72.8% (*p* = 0.77). Five‐year disease‐free survival was 62.1% versus 65.2% (*p* = 0.31) and five‐year cumulative incidence of peritoneal metastases was 24.4% Versus 9.5% (*p* < 0.001). Cytoreductive surgery with HIPEC for peritoneal recurrence was performed in 28 (13.9%) versus 7 patients (3.5%; *p* < 0.001).

**Conclusion:**

Despite increased detection and more frequent treatment of peritoneal metastases in the COLOPEC trial, 5‐year overall and disease‐free survival were comparable to routine practice.

**What does this paper add to the literature?**

This study addresses the uncertainty surrounding the benefits of early detection and treatment of peritoneal metastases in advanced colon cancer. By comparing outcomes between the randomized COLOPEC trial and routine practice using propensity score matching, we demonstrated that despite higher detection and treatment of peritoneal metastases, survival outcomes remain comparable.

## INTRODUCTION

Patients presenting with locally advanced colon cancer with peritoneal invasion or tumour‐related infective complications like tumour perforation are at high risk of developing metachronous peritoneal metastases (PM) following curative resection. Despite the routine use of adjuvant systemic therapy, reported incidences of PM range from 20% to 30% in this high‐risk patient population [1, 2, 3, 4, 5, 6, 7]. Prognosis of patients with PM remains poor and only a minority is eligible for treatment with curative intent. Such treatment consists of cytoreductive surgery (CRS) in specialized centres, which is often combined with hyperthermic intraperitoneal chemotherapy (HIPEC) [8, 9].

Several cohort studies suggested that adjuvant intraperitoneal chemotherapy may reduce the risk of developing PM and potentially improve survival [10]. To verify this hypothesis, the randomized controlled COLOPEC trial evaluated the efficacy of adjuvant oxaliplatin‐based HIPEC following curative resection of locally advanced colon cancer (T4 or perforated tumours) [11]. In this trial, no significant differences in peritoneal metastasis‐free survival (PMFS), disease‐free survival (DFS) or overall survival (OS) were observed between the control and experimental arm [12]. Surprisingly, 9% of patients in the experimental arm already had PM at surgical exploration prior to administration of the adjuvant HIPEC 5–8 weeks after primary tumour resection. These patients remained in the intention‐to‐treat analysis and were considered for therapeutic CRS‐HIPEC if indicated according to the national guideline. The COLOPEC trial also incorporated a diagnostic laparoscopy after a negative CT scan at 18 months in both arms to determine the primary endpoint (peritoneal metastasis‐free survival (PMFS)). Detected peritoneal recurrences were again considered for treatment with CRS‐HIPEC. It was hypothesized that earlier detection of PM with possible curative intent treatment may have improved outcomes in the control arm compared to routine clinical practice, potentially explaining why COLOPEC could not demonstrate a survival benefit of adjuvant oxaliplatin‐based HIPEC.

The impact of intensified follow‐up in both study arms of the COLOPEC trial on long‐term outcomes remains unclear. In this study, we aimed to assess whether long‐term outcomes in the total COLOPEC population differed from that of a comparable cohort of patients with T4 or perforated colon cancer treated outside the trial setting according to routine clinical practice.

## METHODS

### Study design and patient selection

The complete study design and inclusion and exclusion criteria of the COLOPEC trial have been described in detail previously (registered at ClinicalTrials.gov, NCT02231086) [11, 13]. To summarize, between 1 April 2015 and 20 February 2017, patients with clinical or pathological T4 colon cancer or perforated colon cancer were randomized (1:1) to the experimental arm (receiving adjuvant oxaliplatin‐based HIPEC followed by standard adjuvant systemic chemotherapy) or the control arm (receiving only standard adjuvant systemic chemotherapy). For the present analysis, patients from both arms of the COLOPEC trial were included as a single cohort and in this study compared as a whole against the matched control group.

An extensively documented multicentre colon cancer cohort from the Dutch Snapshot Research Group (DSRG) was used to create the control group [14]. In short, patients who underwent curative resection for colon cancer between 1 January 2014 and 31 December 2015 were identified through a mandatory nationwide registry, with extraction of available data. Patients received treatment and routine follow‐up according to the Dutch national guidelines, which included adjuvant chemotherapy when indicated (capecitabin and oxaliplatin (CAPOX), 5‐FU and oxaliplatin (FOLFOX) or capecitabine monotherapy) for a total of 6 months, periodic CEA measurements (3–12 months intervals) and imaging with liver ultrasound and chest X‐ray or CT scan (6–12 months intervals) during the first 5 years. Additional clinical data on tumour characteristics, treatment and long‐term outcomes were collected in three consecutive rounds by collaborators from 50 Dutch hospitals between October 2021 and June 2024, after which all registered data were merged and anonymized.

To create the control group for this study, the inclusion criteria of the COLOPEC trial were applied on the DSRG cohort. Patients who underwent curative intent resection of a colon cancer with cT4/pT4 category or perforation were included. Patients with synchronous metastases were excluded. Since the two cohorts had an overlapping timeframe of 9 months, patients who participated in the COLOPEC trial were excluded from the control cohort.

Ethical approval for the individual trials has previously been obtained (MEC AMC Amsterdam, The Netherlands, 2014‐264, NL49960.018.14; Zwolle, the Netherlands; approval number 210512). All participants in the COLOPEC trial provided informed consent, whereas informed consent was waived for the retrospective identification and data collection of the control cohort. For conducting the present study with re‐use of two existing databases, new ethical approval was obtained (Erasmus MC, MEC‐2025‐0482). This study adhered to The STROBE (Strengthening the Reporting of Observational Studies in Epidemiology) guidelines in the study protocol and manuscript [15].

### Outcome parameters

The primary outcome parameter was 5‐year OS. OS was defined as the time from primary tumour resection until death from any cause. In case no event occurred, patients were censored at last date of follow‐up. Secondary outcome parameters included 5‐year DFS, 5‐year cumulative incidence of PM and proportion of patients treated for PM with curative intent (CRS‐HIPEC). DFS was defined as time from primary tumour until any type of recurrence (locoregional, peritoneal or distant) or death. In case no event occurred, patients were censored at last date of follow‐up. PM was defined as tumour spread to the peritoneum, omentum and/or adnex(a)/ovaries.

### Statistical analysis

All analyses were performed using R software (version 4.3.2) [16]. A propensity score matched analysis was performed to compare patients of both arms of the COLOPEC trial to the control cohort. No formal sample size calculation was performed for this comparative analysis. The available sample size was determined by the number of patients included in the COLOPEC trial and the possibility to find appropriate matched control patients. The matching variables were based on risk factors for developing PM or influencing oncological outcomes, including age, ASA score, pathological T and N categories, tumour side, surgical approach for resection of the primary tumour, presence of perforation, resection margin status and administration of adjuvant chemotherapy. Any missing data were imputed before matching using multiple imputation by chained equations.

Nearest neighbour matching with a ratio of 1:1 was performed without replacement, with a calliper of 0.2 logit to the standard deviation of the propensity score, considered for optimal matching. Love and density plots were performed to visualize the propensity scores. The balance of variables of the matched cohort was assessed using standardized mean differences. Standardized mean differences were calculated as the difference in means between the two groups divided by the square root of two pooled variance of the groups.

Median follow‐up time was estimated using the reverse Kaplan–Meier method, with time to last follow‐up considered the event and deaths censored. OS and DFS were assessed using Kaplan–Meier survival curves and compared using cox proportional hazards model. Clustering was handled by stratifying in the model based on the matched pairs. Cumulative incidence of PM was assessed using a competing risk analysis with a Grey's test, in which the competing risk was death [17].

## RESULTS

### Study population and matching

**FIGURE 1:**
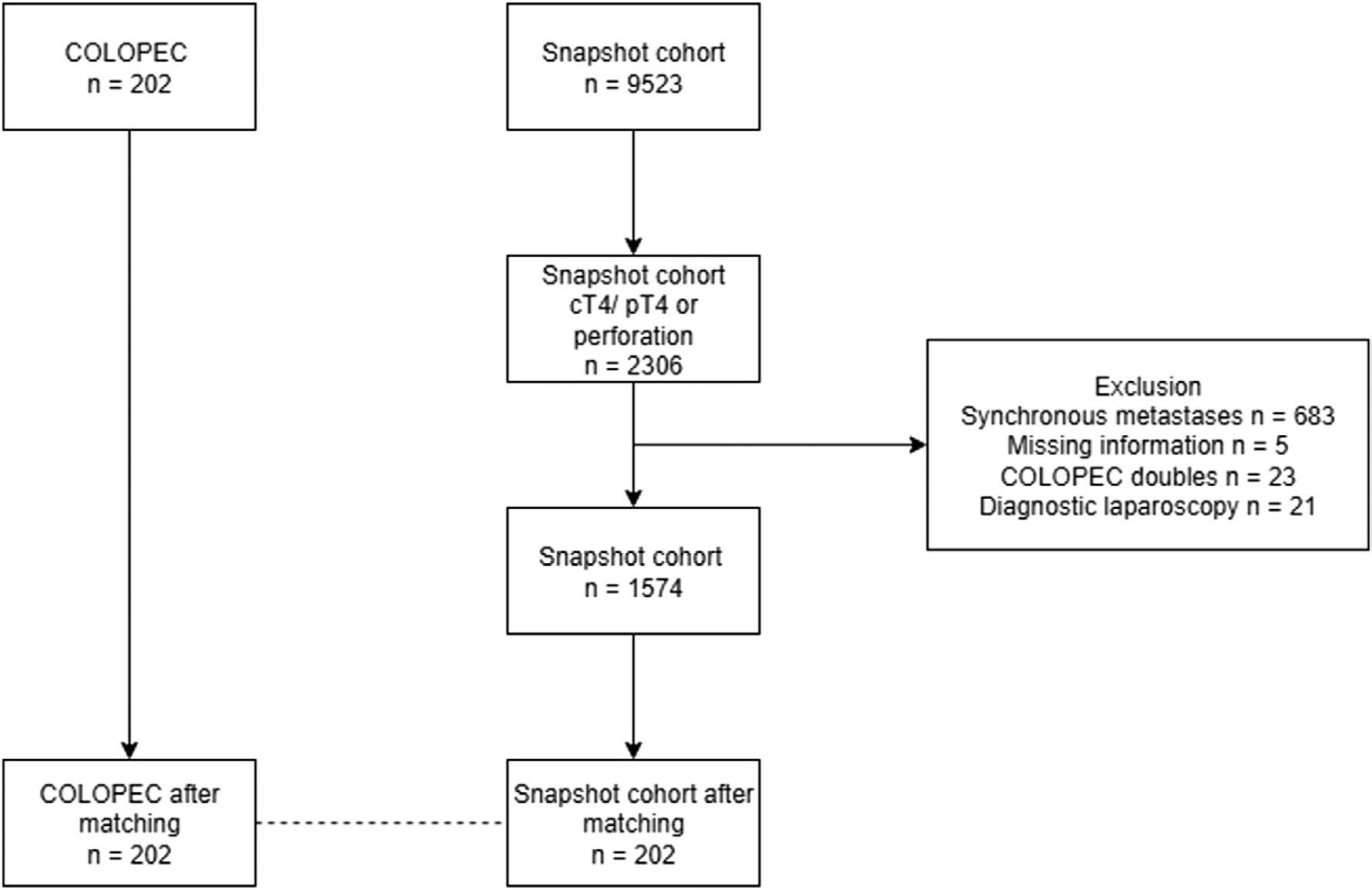
Flow diagram.

Originally, 202 patients were included in the COLOPEC trial and 9,523 patients in the control cohort. After applying COLOPEC trial inclusion criteria and exclusion of trial participants, the control cohort consisted of 1,574 patients (Figure 1) and was used for propensity score matching. Before propensity score matching, patients in the control cohort were older, had higher ASA classifications and BMI, and more frequently underwent emergency surgery with an open approach. They also presented more often with right‐sided tumours and perforations, underwent more often multivisceral resections and had more often positive resection margins, while pT and pN categories were lower compared with patients from the COLOPEC trial. All patients of the COLOPEC trial were included in the analysis. Propensity score matching in a 1:1 ratio resulted in two groups of each 202 patients with well‐balanced baseline characteristics (Table 1). Matching in higher ratios (1:2 or 1:3) resulted in persistently high standardized mean differences and was therefore not pursued. Density plots of the estimated propensity scores before and after 1:1 matching are shown in Figure S1.

**TABLE 1:** Baseline characteristics before and after propensity score matching.

	Before matching			After matching		
	COLOPEC (*n* = 202)	Control (*n* = 1574)	SMD	COLOPEC (*n* = 202)	Control (*n* = 202)	SMD
Sex, *n* (%)						
Female	97 (48.0)	845 (53.7)	0.114	97 (48.0)	98 (48.5)	0.010
Age, *n* (%)						
< 60 years	84 (41.6)	228 (14.5)	1.199	84 (41.6)	73 (36.1)	0.125
60–75 years	115 (56.9)	682 (43.3)		115 (56.9)	127 (62.9)	
> 75 years	3 (1.5)	664 (42.2)		3 (1.5)	2 (1.0)	
ASA score, *n* (%)						
1	82 (40.8)	252 (16.0)	0.819	83 (41.1)	67 (33.2)	0.172
2	111 (55.2)	885 (56.3)		111 (55.0)	128 (63.4)	
3	8 (4.0)	398 (25.3)		8 (4.0)	7 (3.5)	
4	0 (0.0)	36 (2.3)		0 (0.0)	0 (0.0)	
5	0 (0.0)	1 (0.1)		0 (0.0)	0 (0.0)	
BMI, *n* (%)						
< 18.5 kg/m2	3 (1.5)	46 (2.9)	0.161	3 (1.5)	5 (2.5)	0.177
18.5–24.9 kg/m^2^	99 (49.0)	663 (42.1)		101 (50.0)	84 (41.6)	
> 24.9 kg/m2	95 (47.0)	818 (52.0)		98 (48.5)	113 (55.9)	
Setting, *n* (%)						
Elective	160 (80.0)	1185 (75.3)	0.113	161 (79.7)	163 (80.7)	0.025
Emergency	40 (20.0)	389 (24.7)		41 (20.3)	39 (19.3)	
Procedure, *n* (%)						
(Extended) Right hemicolectomy	76 (38.0)	804 (51.1)	0.347	78 (38.6)	83 (41.1)	0.061
(Extended) Left hemicolectomy	26 (13.0)	180 (11.4)		26 (12.9)	27 (13.4)	
Sigmoid resection	94 (47.0)	526 (33.4)		94 (46.5)	88 (43.6)	
Subtotal colectomy	4 (2.0)	41 (2.6)		4 (2.0)	4 (2.0)	
Other	0 (0.0)	23 (1.5)		0 (0.0)	0 (0.0)	
Perforation, *n* (%)						
Perforation	41 (20.4)	277 (17.6)	0.071	42 (20.8)	40 (19.8)	0.025
No perforation	160 (79.6)	1297 (82.4)		160 (79.2)	162 (80.2)	
Localization, *n* (%)						
Right	88 (43.8)	851 (54.1)	0.207	89 (44.1)	92 (45.5)	0.030
Left	113 (56.2)	723 (45.9)		113 (55.9)	110 (54.5)	
Approach, *n* (%)						
Open	70 (35.0)	783 (49.8)	0.365	72 (35.6)	80 (39.6)	0.097
Laparoscopic	110 (55.0)	584 (37.1)		110 (54.5)	106 (52.5)	
Conversion	20 (10.0)	206 (13.1)		20 (9.9)	16 (7.9)	
Multivisceral resection, *n* (%)	70 (34.8)	673 (42.8)	0.165	71 (35.1)	58 (28.7)	0.138
Residual, *n* (%)						
R0	198 (98.0)	1023 (65.1)	0.956	198 (98.0)	197 (97.5)	0.038
R1	3 (1.5)	99 (6.3)		3 (1.5)	4 (2.0)	
R2	1 (0.5)	28 (1.8)		1 (0.5)	1 (0.5)	
Rx	0 (0.0)	422 (26.8)		0 (0.0)	0 (0.0)	
pT category, *n* (%)						
pT1	0 (0.0)	11 (0.7)	0.481	0 (0.0)	0 (0.0)	0.059
pT2	2 (1.0)	29 (1.8)		2 (1.0)	2 (1.0)	
pT3	24 (11.9)	467 (29.7)		24 (11.9)	28 (13.9)	
pT4	175 (87.1)	1066 (67.8)		176 (87.1)	172 (85.1)	
pN category, *n* (%)						
pN0	53 (26.4)	767 (48.7)	0.517	54 (26.7)	58 (28.7)	0.050
pN1	70 (34.8)	480 (30.5)		70 (34.7)	70 (34.7)	
pN2	78 (38.8)	326 (20.7)		78 (38.6)	74 (36.6)	
pNx	0 (0.0)	1 (0.1)		0 (0.0)	0 (0.0)	
Histopathology, *n* (%)						
Adenocarcinoma, well to moderately differentiated	147 (73.1)	1100 (71.2)	0.210	147 (72.8)	151 (74.8)	0.198
Adenocarcinoma, poorly differentiated	25 (12.4)	209 (13.5)		26 (12.9)	28 (13.9)	
Mucinous adenocarcinoma	17 (8.5)	181 (11.7)		17 (8.4)	18 (8.9)	
Signet ring cell carcinoma	10 (5.0)	29 (1.9)		10 (5.0)	3 (1.5)	
Other	2 (1.0)	26 (1.7)		2 (1.0)	2 (1.0)	
Adjuvant chemotherapy, *n* (%)	175 (87.1)	652 (41.5)	1.080	175 (86.6)	183 (90.6)	0.125
Type of adjuvant chemotherapy, *n* (%)						
CAPOX or FOLFOX	162 (92.6)	501 (76.8)	0.600	162 (92.6)	161 (88.0)	0.226
Capecitabine monotherapy	2 (1.1)	118 (18.1)		2 (1.1)	9 (4.9)	
Unknown	11 (6.3)	33 (5.1)		11 (6.3)	13 (7.1)	
Duration of chemotherapy, median (IQR) in days^a^	168 (126–168)	138 (114–170)	0.233	168 (126–168)	156 (128–175)	0.030
Missing	4	315		4	109	

Abbreviations: ASA, American Society of Anesthesiologists Classification; BMI, body mass index; IQR, interquartile range; SMD, standardized mean difference.

^a^ In the COLOPEC cohort, only the number of chemotherapy courses was available. As a course of CAPOX or capecitabine monotherapy lasts 21 days and a course of FOLFOX lasts 14 days, duration in days was estimated by multiplying the number of courses by the corresponding course length.

### Oncological outcomes

**FIGURE 2:**
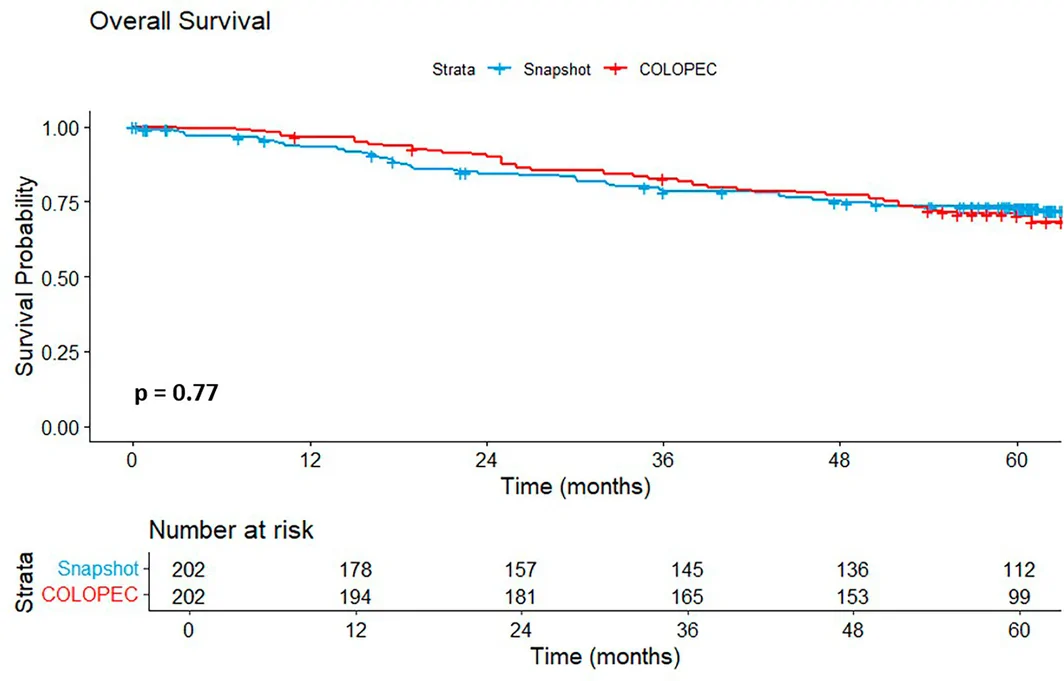
Overall survival of the matched COLOPEC and control cohorts.

**FIGURE 3:**
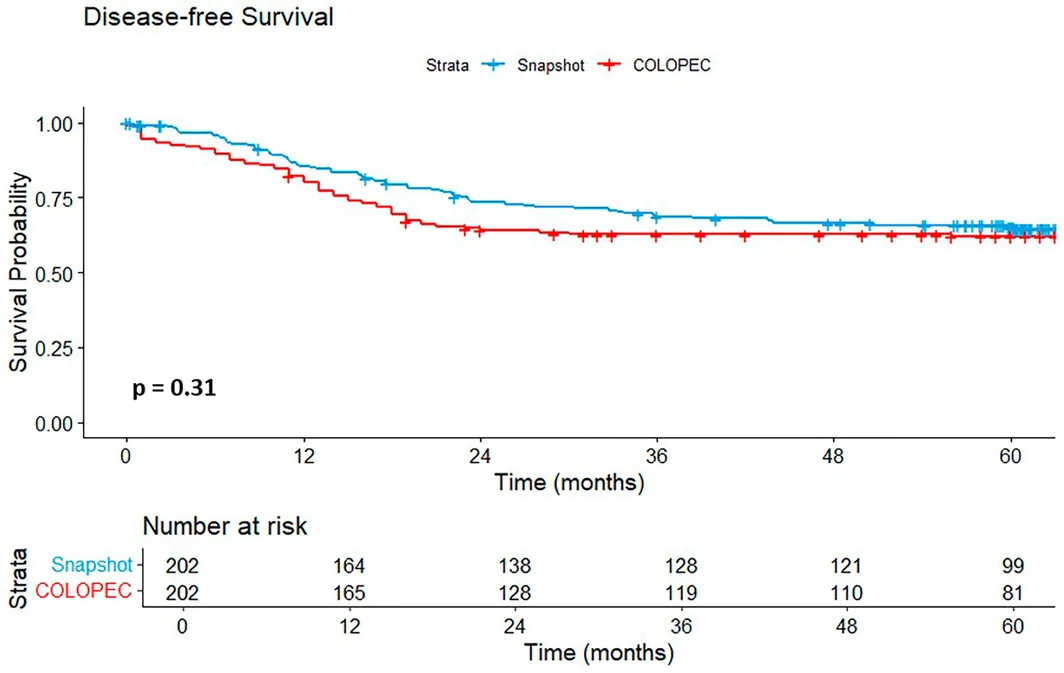
Disease‐free survival of the matched COLOPEC and control cohorts.

Median follow‐up was 60 months (IQR 59–62) in the COLOPEC trial and 64 months (IQR 60–82) in the matched control cohort. Five‐year OS of patients included in the COLOPEC trial was 70.2%, and 5‐year OS was 72.8% in the matched control cohort (*p* = 0.77) (Figure 2). Five‐year DFS was neither significantly different between patients of the COLOPEC trial and the matched control cohort: 62.1% versus 65.2%, respectively (*p* = 0.31) (Figure 3). OS and DFS Kaplan–Meier curves before matching are presented in Figures S2 and S3.

### Cumulative incidence peritoneal metastases and treatment

**FIGURE 4:**
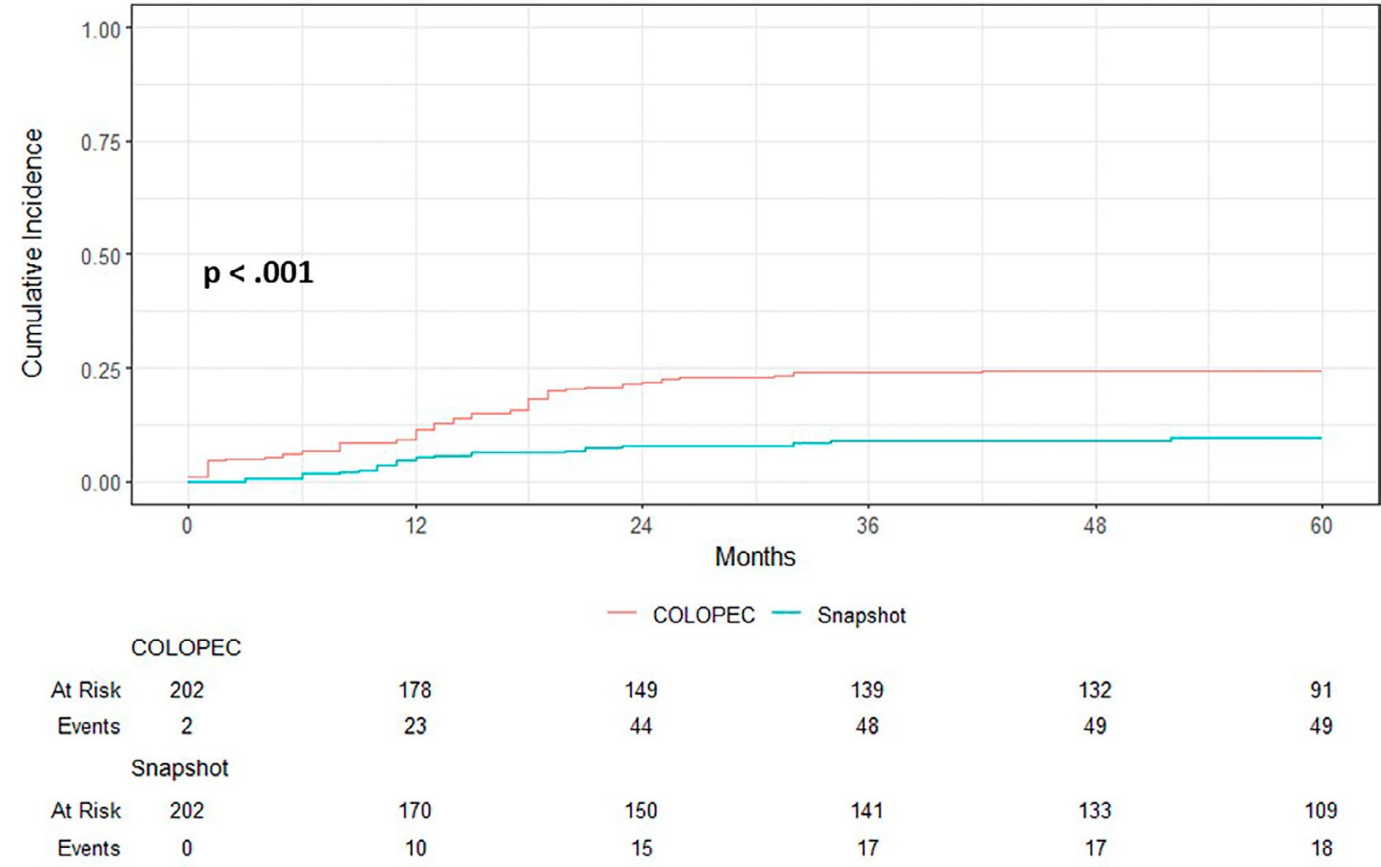
Cumulative incidence of peritoneal metastases of the matched COLOPEC and control cohorts.

Five‐year cumulative incidence of PM in the COLOPEC trial was 24.4% (95% CI 18.7–30.5), compared to 9.5% (95% CI 5.9–14.2) in the matched control cohort (*p* < 0.001) (Figure 4). Of patients diagnosed with PM, 28 (57.1%) received CRS‐HIPEC in the COLOPEC trial compared to 7 (38.9%) in the matched control cohort. The proportions of patients who underwent CRS‐HIPEC from the overall patient groups were 13.9% and 3.5% (*p* < 0.001), respectively.

## DISCUSSION AND CONCLUSIONS

In this propensity score matched analysis between the COLOPEC trial and a matched control cohort, no differences were observed in 5‐year OS and 5‐year DFS, while more intensive follow‐up including laparoscopic re‐explorations in the COLOPEC trial resulted in a significantly higher incidence of PM, as well as a substantially higher proportion of patients who underwent curative CRS‐HIPEC. These findings demonstrate that intensified follow‐up in high‐risk patient populations, including the use of diagnostic laparoscopy, increases the detection and treatment of occult PM, but this does not seem to translate into improved survival.

The current results align with previous studies that determined the impact of more intensified follow‐up regimens on long‐term outcomes in different fields of oncology, which similarly demonstrated that intensified follow‐up results in more salvage treatment, but not a survival benefit [18, 19, 20]. These findings support the hypothesis that early detection of recurrence does not alter the natural course of disease in at least colorectal cancer patients, including those within a selected high‐risk population in the present study.

The difference in incidence of PM between the COLOPEC trial and the matched control cohort is likely caused by the intensified follow‐up with a specific focus on occult PM, rather than differences in disease stage. This interpretation is supported by the high success of matching with low standardized mean differences for key variables, such as pathological T and N categories, and the presence of perforation. Nevertheless, residual differences in tumour biology cannot be completely ruled out since data on for example molecular characteristics were unavailable. Despite this limitation, the comparable 5‐year DFS between groups, which incorporates all recurrence types, provides indirect reassurance that overall disease burden at baseline was broadly similar. Routine follow‐up in the Netherlands at that time often included abdominal ultrasound, but even abdominal CT has a limited sensitivity to detect PM [21, 22]. This was the reason to incorporate diagnostic laparoscopy into the follow‐up schedule of both the experimental and control arm of the COLOPEC trial, which could reliably assess the primary end‐point by also detecting small peritoneal recurrences that were missed on CT. In the control cohort representing routine practice outside trial setting, most PM remained undetected, even after 5‐years of follow‐up, which might be explained by a focus on liver, lung, and other locations in the metastatic setting. Response to systemic therapy is often evaluated based on RECIST (Response Evaluation Criteria in Solid Tumours) criteria, using clearly visible and measurable metastases such as liver metastases [23]. In addition, in routine clinical practice once incurable metastatic disease is diagnosed, active surveillance for additional metastatic sites—such as peritoneal metastases—generally ceases when patients transition to palliative or best supportive care, whereas trial protocols typically continue systematic assessment. The comparable 5‐year DFS—in which peritoneal, locoregional and distant metastases were considered—provided a certain degree of confidence that proper oncological matching was achieved, with a similar proportion of distant spread, and only a difference in PM related to specific focused follow‐up.

This study also demonstrated the problem of generalizability of randomized clinical trial outcomes. Although randomized clinical trials are essential for their controlled conditions, results may not be extrapolated to populations outside the trial setting. Trial participants are often younger with less comorbidity compared to the general patient population, as was demonstrated in this study. This can limit the external validity. Despite propensity score matching on key baseline characteristics, there might be residual confounding, as patients who consented to participate in randomized trials often represent a distinct group with different, unmeasurable confounding characteristics. Nevertheless, the use of propensity score matching allowed for a balanced comparison between COLOPEC trial participants and real‐world data. The ratio of 1:1 matching ensured inclusion of all patients and avoided selection bias related to unmatched patients.

However, certain limitations remain. Registration of recurrence patterns and oncological follow‐up in both the control cohort and COLOPEC trial rely on how this was documented in the original patient files, and is influenced by the heterogeneity of follow‐up regimens applied by the different hospitals. For example, the type and duration of adjuvant systemic therapy were not known for all patients in the control cohort, and for the COLOPEC cohort, only the number of chemotherapy courses given was known rather than treatment duration directly. Nevertheless, by converting the number of courses to an estimated duration in days, comparable data on chemotherapy could be obtained for both cohorts. For overall survival, however, underreporting is unlikely, as mortality data in the Netherlands are reliably registered through national registries with high completeness of follow‐up. Finally, there is always the risk of residual confounding despite propensity score matching, which should be taken into account when interpreting the current findings.

Regarding future research, the COLOPEC II trial will provide important additional evidence on the role of intensified follow‐up [24]. High‐risk patients routinely received a second look laparoscopy at 6 months, and were randomized between standard of care follow‐up and a third look laparoscopy at 18 months.

To conclude, several focused interventions on prevention, early diagnosis and treatment of PM as investigated in the COLOPEC trial seemed not to improve patient survival compared to standard clinical practice. Abdominal re‐exploration resulted in substantially more and early detection of PM, leading to more salvage treatments with associated morbidity. These findings should initiate critical appraisal of the underlying concepts and their consequences for clinical practice.

## AUTHOR CONTRIBUTIONS

**Iris Hochstenbach:** Conceptualization; data curation; formal analysis; methodology; project administration; validation; investigation; writing – original draft; writing – review and editing. **Julie J. M. Hamm:** Conceptualization; data curation; formal analysis; methodology; project administration; validation; investigation; writing – original draft; writing – review and editing. **Eva Rademaker:** Conceptualization; data curation; methodology; validation; investigation; writing – review and editing. **Arend G. J. Aalbers:** investigation; Writing – review and editing. **Djamila Boerma:** Investigation; writing – review and editing. **Wilhelmina M. U. van Grevenstein:** Investigation; writing – review and editing. **Patrick H. J. Hemmer:** Investigation; writing – review and editing. **Ignace H. J. T. De Hingh:** Investigation; writing – review and editing. **Niels F. M. Kok:** Investigation; writing – review and editing. **Jurriaan B. Tuynman:** Investigation; writing – review and editing. **Eva V. E. Madsen:** Investigation; writing – review and editing. **Johannes H. W. de Wilt:** Investigation; writing – review and editing. **Esther C. J. Consten:** Conceptualization; methodology; supervision; investigation; writing – review and editing. **Henderik L. van Westreenen:** Conceptualization; funding acquisition; methodology; supervision; writing – review and editing. **Pieter J. Tanis:** Conceptualization; data curation; funding acquisition; methodology; supervision; validation; investigation; writing – review and editing.

## FUNDING INFORMATION

The COLOPEC trial was funded by the Netherlands Organization for Health Research and Development (ZonMW) and the Dutch cancer society (KWF). The Snapshot complex colon cancer study was funded by the Dutch Cancer Society (KWF) and the Isala Academy for Innovation and research.

## CONFLICT OF INTEREST STATEMENT

The authors declare no conflict of interest.

## ETHICS STATEMENT

This study was approved by the Erasmus MC Medical Ethics Committee (Rotterdam, the Netherlands, MEC‐2025‐0482). Ethical approval for the individual trials has previously been obtained (MEC AMC Amsterdam, The Netherlands, 2014‐264, NL49960.018.14; Zwolle, the Netherlands; approval number 210512). All participants in the COLOPEC trial provided written informed consent, whereas informed consent was waived for the retrospective identification and data collection of the control cohort. The study is performed in accordance with the Declaration of Helsinki.

## Supporting information


**Figure S1.** Density plot distribution of estimated propensity scores before and after matching.
**Figure S2.** Overall survival before matching.
**Figure S3.** Disease‐free survival before matching.

**Data S1.** STROBE statement—Checklist of items that should be included in reports of case–control studies.

## Data Availability

Individual de‐identified data underlying this study's findings can be obtained upon reasonable request from the corresponding author with a signed data transfer agreement and for scientific purposes only.

## APPENDIX A

### COLLABORATORS LISTS

**COLOPEC collaborators group** Caroline S. Andeweg (Hospital St Jansdal, Department of Surgery, Lelystad, the Netherlands), Vivian P. Bastiaenen (Amsterdam University Medical Center, location University of Amsterdam, Department of Surgery, Amsterdam, the Netherlands), Willem A. Bemelman (Amsterdam University Medical Center, location University of Amsterdam, Department of Surgery, Amsterdam, the Netherlands), Jarmila D.W. van der Bilt (Amsterdam University Medical Center, location University of Amsterdam, Department of Surgery, Amsterdam, the Netherlands), Johanne Bloemen (Catharina Hospital, Department of Surgery, Eindhoven, the Netherlands), Frank C. den Boer (Deceased) (Zaans Medical Center, Department of Surgery, Zaandam, the Netherlands), Daan ten Bokkel Huinink (Alexander Monro Hospital, Cancer Clinic, Bilthoven, the Netherlands), Alexandra R.M. Brandt‐Kerkhof (Erasmus Medical Center, Department of Surgical Oncology and Gastrointestinal Surgery, Rotterdam, the Netherlands), Andre J.A. Bremers (Radboud University Medical Center, Department of Surgery, Nijmegen, the Netherlands), Walter J.A. Brokelman (Jeroen Bosch Hospital, Department of Surgery, s‐Hertogenbosch, the Netherlands), Huib A. Cense (Rode Kruis Hospital, Department of Surgery, Beverwijk, the Netherlands), Geert‐Jan Creemers (Catharina Hospital, Department of Surgery, Eindhoven, the Netherlands), Johannes Crezee (Amsterdam University Medical Center, location University of Amsterdam, Department of Radiation Oncology, Amsterdam, the Netherlands), Rogier M.P.H. Crolla (Amphia Hospital, Department of Surgery, Breda, the Netherlands), Jan‐Willem T. Dekker (Reinier De Graaf Hospital, Department of Surgery, Delft, the Netherlands), Jennifer Demelinne (Catharina Hospital, Department of Surgery, Eindhoven, the Netherlands), Marc J. van Det (Ziekehuisgroep Twente, Department of Surgery, Hengelo, the Netherlands), Karin K. van Diepen (University Medical Center Utrecht, Department of Surgery, Utrecht, the Netherlands), Marjolein Diepeveen (Erasmus Medical Center, Department of Surgical Oncology and Gastrointestinal Surgery, Rotterdam, the Netherland), Marcel G.W. Dijkgraaf (Amsterdam University Medical Center, location University of Amsterdam, Department of Epidemiology and Data Science, Amsterdam, the Netherlands), Eino B. van Duyn (Medisch Spectrum Twente, Department of Surgery, Enschede, the Netherlands), Esther D. van den Ende (Ziekehuisgroep Twente, Department of Surgery, Hengelo, the Netherlands), Pauline Evers (Nederlandse Federatie van Kankerpatientorganisaties (NFK), DT, Utrecht, the Netherlands), Hans F.J. Fabry (Bravis Hospital, Department of Surgery, Roosendaal, the Netherlands), Floris T.J. Ferenschild (Maashospital Pantein, Department of Surgery, Beugen, the Netherlands), Sebastiaan Festen (Onze Lieve Vrouwen Gasthuis, Department of Surgery, Amsterdam, the Netherlands), Anna A.W. van Geloven (TerGooi Medical Center, Department of Surgery, Hilversum, the Netherlands), Erwin van der Harst (Maasstad Hospital, Department of Surgery, Rotterdam, the Netherlands), Jeroen Heemskerk (Laurentius Hospital, Department of Surgery, Roermond, the Netherlands), Joost T. Heikens (Jeroen Bosch Hospital, Department of Surgery, ‘s‐Hertogenbosch, the Netherlands), Daniel A. Hess (Antonius Hospital, Department of Surgery, Sneek, the Netherlands), Bas Inberg (Streekziekenhuis Koningin Beatrix, Department of Surgery, Winterswijk, the Netherlands), Jan Jansen (ADRZ, Department of Surgery, Zierikzee, the Netherlands), Charlotte E.L. Klaver (Amsterdam University Medical Center, location University of Amsterdam, Department of Surgery, Amsterdam, the Netherlands), Frank W.H. Kloppenberg (Treant Zorggroep, Department of Surgery, Emmen, the Netherlands), Thomas J.M. Kootstra (St. Antonius Hospital, Department of Surgery, Nieuwegein, the Netherlands), R.T.J. Kortekaas (Franciscus Gasthuis & Vlietland, Department of Surgery, Rotterdam, the Netherlands), Maartje Los (St. Antonius Hospital, Department of Surgery, Nieuwegein, the Netherlands), H.C.J. van der Mijle (Medical Center Leeuwarden, Department of Surgery, Leeuwarden, the Netherlands), Linda Mol (Integraal Kankercentrum Nederland, Utrecht, the Netherlands), Gijsbert D. Musters (Amsterdam University Medical Center, location University of Amsterdam, Department of Surgery, Amsterdam, the Netherlands), Peter A. Neijenhuis (Alrijne hospital, Department of Surgery, Leiden, the Netherlands), Simon W. Nienhuijs (Catharina Hospital, Department of Surgery, Eindhoven, the Netherlands), Loes van den Nieuwenhof (Catharina Hospital, Department of Surgery, Eindhoven, the Netherlands), Koen C.M.J. Peeters (Leiden University Medical Center, Department of Surgery, Leiden, the Netherlands), Sebastiaan W. Polle (Amsterdam University Medical Center, location University of Amsterdam, Department of Surgery, Amsterdam, the Netherlands), Jolien Pon, Pieter Poortman (Dijklander Hospital, Department of Surgery, Hoorn, the Netherlands), Cornelis J.A. Punt (Amsterdam University Medical Center, location University of Amsterdam, Department of Oncology, Amsterdam, the Netherlands), Sandra A. Radema (Radboud Oncologie Fonds, Nijmegen, the Netherlands), Bert van Ramshorst (St. Antonius Hospital, Department of Surgery, Nieuwegein, the Netherlands), Philip R. de Reuver (Radboud University Medical Center, Department of Surgery, Nijmegen, the Netherlands), Koen P. Rovers (Catharina Hospital, Department of Surgery, Eindhoven, the Netherlands), Roderick F. Schmitz (Groene Hart Hospital, Department of Surgery, Gouda, the Netherlands), Lotte Schoonderwoerd (Bernhoven Hospital, Department of Surgery, Uden, the Netherlands), Nina Sluiter (Amsterdam University Medical Center, Free University, Department of Surgery, Amsterdam, the Netherlands), Dirkje W. Sommeijer (Amsterdam University Medical Center, location University of Amsterdam, Department of Surgery, Amsterdam, the Netherlands), Eric Sonneveld (Dijklander Hospital, Department of Surgery, Hoorn, the Netherlands), T.C. van Sprundel (Ommelander Ziekenhuis Groningen, Department of Surgery, Scheemda, the Netherlands), Sanne C. Veltkamp (Amstelland Hospital, Department of Surgery, Amstelveen, the Netherlands), Maarten Vermaas (Ijsselland Hospital, Department of Surgery, Capelle aan den Ijssel, the Netherlands), Victor J. Verwaal (Catharina Hospital, Department of Surgery, Eindhoven, the Netherlands), Emma Wassenaar (St. Antonius Hospital, Department of Surgery, Nieuwegein, the Netherlands), Johannes A. Wegdam (Elkerliek Hospital, Department of Surgery, Helmond, the Netherlands), Marinke Westerterp (Netherlands Cancer Institute/Antoni van Leeuwenhoek Hospital, Amsterdam, the Netherlands), Marinus J. Wiezer (St Antonius Hospital, Department of Surgery, Nieuwegein, the Netherlands), Daniel D. Wisselink (Amsterdam University Medical Center, location University of Amsterdam, Department of Surgery, Amsterdam, the Netherlands), Fennie Wit (Tjongerschans hospital, Department of Surgery, Heerenveen, the Netherlands), Arjen J. Witkamp (University Medical Center Utrecht, Department of Surgery, Utrecht, the Netherlands), Karlijn van Woensdregt (Hospital St Jansdal, Department of Surgery, Lelystad, the Netherlands), Edwin S. van der Zaag (Gelre Hospital, Department of Surgery, Apeldoorn, the Netherlands), David D.E. Zimmerman (Elisabeth‐Tweesteden Hospital, Department of Surgery, Tilburg, the Netherlands), Mandy Zournas (St. Antonius Hospital, Department of Surgery, Nieuwegein, the Netherlands), Annette van Zweeden (Amstelland Hospital, Department of Internal Medicine, Amstelveen, the Netherlands).

**Dutch Snapshot Research Group** D. C. Van der Aa (Amsterdam University Medical Center, Amsterdam, the Netherlands), S. M. Van Aalten (Groene Hart Hospital, Gouda, the Netherlands), I. Aanen (Haga Hospital, The Hague, the Netherlands), F. J. Amelung (Jeroen Bosch Hospital, Den Bosch, the Netherlands), P. Van Amstel (Flevo Hospital, Almere, the Netherlands), E. Ancion (Zuyderland Medical Center, Heerlen and Sittard/Geleen, the Netherlands), J. J. Atema (Onze Lieve Vrouwen Gasthuis, Amsterdam, the Netherlands), T. S. Aukema (Haga Hospital, the Hague, the Netherlands), V. Baart (Groene Hart Hospital, Gouda, the Netherlands), E. Z. Barsom (Amsterdam University Medical Center, Amsterdam, the Netherlands), V. P. Bastiaenen (Flevo Hospital, Almere, the Netherlands), T. Bazuin (Treant, Emmen, the Netherlands), M. A. J. Becker (Flevo Hospital, Almere, the Netherlands), V. M. Belvroy (Franciscus Gasthuis, Rotterdam, the Netherlands), R. H. A. Berndsen (Spaarne Gasthuis, Hoofddorp, the Netherlands), B. Biersteker (Groene Hart Hospital, Gouda, the Netherlands), J. D. W. Van der Bilt (Flevo Hospital, Almere, the Netherlands), J. G. Bloemen (Catharina Hospital, Eindhoven, the Netherlands), S. Bluiminck (Radboud University Medical Center, Nijmegen, the Netherlands), F. C. Den Boer† (Zaans Medical Center, Zaandam, the Netherlands), E. G. Boerma (Zuyderland Medical Center, Heerlen and Sittard/Geleen, the Netherlands), M. C. Boonstra (Onze Lieve Vrouwen Gasthuis, Amsterdam, the Netherlands), W. A. A. Borstlap (Albert Schweitzer Hospital, Dordrecht, the Netherlands), L. A. Brugts (Maasstad Hospital, Rotterdam, the Netherlands), J. W. A. Burger (Catharina Hospital, Eindhoven, the Netherlands), T. A. Burghgraef (Meander Medical Center, Amersfoort, the Netherlands), S. M. M. De Castro (Onze Lieve Vrouwen Gasthuis, Amsterdam, the Netherlands), M. Cleveringa (Diakonessenhuis, Utrecht, the Netherlands), R. J. S. Coelen (Dijklander Hospital, Hoorn, the Netherlands), A. Demirkiran (Rode Kruis Hospital, Beverwijk, the Netherlands), E. Dijkema (Slingeland Hospital, Doetinchem, the Netherlands), S. Dijkink (Haaglanden Medical Center, The Hague, the Netherlands), S. A. Dingemans (TergooiMC, Hilversum, the Netherlands), L. Van Dullemen (Martini Hospital, Groningen, the Netherlands), E. B. Van Duyn (Medisch Spectrum Twente, Enschede, the Netherlands), A. J. Eleveld (Treant, Emmen, the Netherlands), H. A. Galema (Erasmus MC, Rotterdam, the Netherlands), E. G. M. Van Geffen (Amsterdam University Medical Center, Amsterdam, the Netherlands), R. T. J. Geitenbeek (University Medical Center Groningen, Groningen, the Netherlands), A. A. W. Van Geloven (TergooiMC, Hilversum, the Netherlands), A. H. C. Gielen (Maastricht University Medical Center, Maastricht, the Netherlands), C. A. Gispen (Leiden University Medical Center, Leiden, the Netherlands), M. J. P. M. Govaert (Dijklander Hospital, Hoorn, the Netherlands), V. P. Groot (Antonius Hospital, Nieuwegein, the Netherlands), B. A. Grotenhuis (Netherlands Cancer Institute/Antoni van Leeuwenhoek Hospital, Amsterdam, the Netherlands), A. A. J. Grüter (Amsterdam University Medical Center, Amsterdam, the Netherlands), S. F. Hardon (Zaans Medical Center, Zaandam, the Netherlands), A. G. Den Hartog (Netherlands Cancer Institute/Antoni van Leeuwenhoek Hospital, Amsterdam, the Netherlands), K. Havenga (University Medical Center Groningen, Groningen, the Netherlands), J. A. G. Van der Heijden (Catharina Hospital, Eindhoven, the Netherlands), F. Heilijgers (Haaglanden Medical Center, The Hague, the Netherlands), T. B. M. Van den Heuvel (Catharina Hospital, Eindhoven, the Netherlands), R. S. Hijmans (Frisius Medical Center, Leeuwarden, the Netherlands), J. Van Hilst (Onze Lieve Vrouwen Gasthuis, Amsterdam, the Netherlands), J. C. Hol (Gelderse Vallei Hospital, Ede, the Netherlands), S. Janki (Haga Hospital, the Hague, the Netherlands), A. C. H. M. Jongen (Catharina Hospital, Eindhoven, the Netherlands), I. Kappers (Tjongerschans, Heerenveen, the Netherlands), S. Karhof (VieCuri Medical Center, Venlo‐Venray, the Netherlands), B. A. J. Kertzman (Jeroen Bosch Hospital, Den Bosch, the Netherlands), M. M. Kieboom (University Medical Center Utrecht, Utrecht, the Netherlands), N. Koemans (Groene Hart Hospital, Gouda, the Netherlands), J. L. M. Konsten (VieCuri Medical Center, Venlo‐Venray, the Netherlands), W. H. Kopp (Antonius Hospital, Nieuwegein, the Netherlands), O. W. Kranenburg (University Medical Center Utrecht, Utrecht, the Netherlands), P. Krielen (Canisius Wilhelmina Hospital, Nijmegen, the Netherlands), A. Kumas (Pantein Hospital, Boxmeer, the Netherlands), M. Kusters (Amsterdam University Medical Center, Amsterdam, the Netherlands), D. P. V. Lambrichts (Reinier de Graaf Gasthuis, Delft, the Netherlands), B. Lamme (Albert Schweitzer Hospital, Dordrecht, the Netherlands), G. L. Van Leeuwen (Martini Hospital, Groningen, the Netherlands), E. W. Lockhorst (Amphia Hospital, Breda, the Netherlands), H. Lutfi (Treant, Emmen, the Netherlands), S. Malm (IJsselland Hospital, Rotterdam, the Netherlands), H. T. J. Mantel (Ziekenhuis Groep Twente, Hengelo, the Netherlands), G. A. Martini (Martini Hospital, Groningen, the Netherlands), P. Meijer (Diakonessenhuis, Utrecht, the Netherlands), J. Melenhorst (Maastricht University Medical Center, Maastricht, the Netherlands), D. M. Mens (Erasmus MC, Rotterdam, the Netherlands), M. J. H. Metman (Maasstad Hospital, Rotterdam, the Netherlands), L. C. F. De Nes (Pantein Hospital, Boxmeer, the Netherlands), L. R. Moolenaar (Amsterdam University Medical Center, Amsterdam, the Netherlands), J. A. Nieuwstraten (Leiden University Medical Center, Leiden, the Netherlands), J. Nonner (Ikazia Hospital, Rotterdam, the Netherlands), S. E. Van Oostendorp (Rode Kruis Hospital, Beverwijk, the Netherlands), S. J. Oosterling (Spaarne Gasthuis, Hoofddorp, the Netherlands), C. M. W. Pesch (Groene Hart Hospital, Gouda, the Netherlands), C. M. L. Peters (Amphia Hospital, Breda, the Netherlands), M. W. H. Philipsen (Zuyderland Medical Center, Heerlen and Sittard/Geleen, the Netherlands), W. Y. Van der Plas (Amsterdam University Medical Center, Amsterdam, the Netherlands), M. M. Poelman (Franciscus Gasthuis, Rotterdam, the Netherlands), F. Polat (Canisius Wilhelmina Hospital, Nijmegen, the Netherlands), A. E. Posma‐Bouman (Slingeland Hospital, Doetinchem, the Netherlands), B. B. Pultrum (Martini Hospital, Groningen, the Netherlands), J. M. Van Rees (Erasmus MC, Rotteradm, the Netherlands), W. P. Reinders (Ziekenhuis Groep Twente, Hengelo, the Netherlands), P. R. De Reuver (Radboud University Medical Center, Nijmegen, the Netherlands), M. C. Richir (Utrecht University Medical Center, Utrecht, the Netherlands), T. W. H. Rijnhout (Pantein Hospital, Boxmeer, the Netherlands), M. M. Romeijn (Maxima Medical Center, Veldhoven, the Netherlands), D. Roorda (Elisabeth‐TweeSteden Hospital, Tilburg, the Netherlands), C. C. Van Rossem (Maasstad Hospital, Rotterdam, the Netherlands), J. Rothbarth (Erasmus MC, Rotterdam, the Netherlands), M. Ruig (Groene Hart Hospital, Gouda, the Netherlands), H. G. Sahin (Flevo Hospital, Almere, the Netherlands), I. Said (Radboud University Medical Center, Nijmegen, the Netherlands), R. A. Schasfoort (Treant, Emmen, the Netherlands), J. Scholten (Spaarne Gasthuis, Hoofddorp, the Netherlands), P. M. E. Schuivens (Rijnstate, Arnhem, the Netherlands), D. Schweitzer (Maastricht University Medical Center, Maastricht, the Netherlands), C. Sietses (Gelderse Vallei Hospital, Ede, the Netherlands), M. Sietzema (Tjongerschans, Heerenveen, the Netherlands), G. D. Slooter (Maxima Medical Center, Veldhoven, the Netherlands), N. Smakman (Diakonessenhuis, Utrecht, the Netherlands), B. P. Smalbroek (Antonius Hospital, Nieuwegein, the Netherlands), E. J. Spillenaar Bilgen (Rijnstate, Arnhem, the Netherlands), E. J. A. Steller (Isala Hospital, Zwolle, the Netherlands), T. F. Stoop (Amsterdam University Medical Center, Amsterdam, the Netherlands), M. Straver (Laurentius Hospital, Roermond, the Netherlands), S. K. Stuart (Rijnstate, Arnhem, the Netherlands), A. Suntharan (Deventer Hospital, Deventer, the Netherlands), A. K. Talsma (Deventer Hospital, Deventer, the Netherlands), K. Trumpi (Diakonessenhuis, Utrecht, the Netherlands), J. Veenhuizen (TergooiMC, Hilversum, the Netherlands), A. W. H. Van de Ven (Flevo Hospital, Almere, the Netherlands), E. G. G. Verdaasdonk (Jeroen Bosch Hospital, Den Bosch, the Netherlands), P. M. Verheijen (Meander Medical Center, Amersfoort, the Netherlands), A. Villerius‐Meijer (Laurentius Hospital, Roermond, the Netherlands), M. Vissers (Laurentius Hospital, Roermond, the Netherlands), E. L. K. Voogt (Franciscus Gasthuis, Rotterdam, the Netherlands), R. P. H. De Vries (Martini Hospital, Groningen, the Netherlands), S. T. Van Vugt (Wilhelmina Hospital, Assen, the Netherlands), D. D. De Waard (University Medical Center, Utrecht, the Netherlands), T. R. Wagner (Zaans Medical Center, Zaandam, the Netherlands), A. K. Warps (Frisius Medical Center, Leeuwarden, the Netherlands), R. H. A. Welling (Elisabeth‐TweeSteden Hospital, Tilburg, the Netherlands), M. Westerterp (Netherlands Cancer Institute/Antoni van Leeuwenhoek Hospital, Amsterdam, the Netherlands), J. K. Wiggers (Amsterdam University Medical Center, Amsterdam, the Netherlands), A. R. Wijsmuller (University Medical Center Groningen, Groningen, the Netherlands), C. D. M. Witjes (IJsselland Hospital, Rotterdam, the Netherlands), B. Zamaray (Amsterdam University Medical Center, Amsterdam, the Netherlands), N. Van Zenden (Ziekenhuis Groep Twente, Hengelo, the Netherlands), S. J. M. Zomer (Rijnstate, Arnhem, the Netherlands), E. S. Zwanenburg (TergooiMC, Hilversum, the Netherlands), E. S. Zwart (Dijklander Hospital, Hoorn, the Netherlands), S. L. M. Zwetsloot (Dijklander Hospital, Hoorn, the Netherlands).

**Dutch Complex Colon Cancer Initiative** M. J. Lahaye (Netherlands Cancer Institute/Antoni van Leeuwenhoek Hospital, Amsterdam, the Netherlands), P. D. Siersema (Erasmus MC, Rotterdam, the Netherlands), P. Snaebjornsson (Netherlands Cancer Institute/Antoni van Leeuwenhoek Hospital, Amsterdam, the Netherlands).
