# Supplementary material for: Detection and treatment of peritoneal metastases in colon cancer: COLOPEC trial compared to routine practice
Source: Colorectal Dis. 2026 Sep 17;28(9):e70614. doi: 10.1111/codi.70614 (PMC13586421; doi:10.1111/codi.70614)
Supplement: Supplementary file 1 [file CODI-28-0-s001.docx]

## Supplemental Digital Content

**Figure S1. Density plot Distribution of Estimated Propensity Scores Before and After Matching**

**Figure S2. Overall Survival Before Matching**

**Figure S3. Disease-free Survival Before Matching**
